# Structural Brain Changes as Biomarkers and Outcome Predictors in Patients with Late-Life Depression: A Cross-Sectional and Prospective Study

**DOI:** 10.1371/journal.pone.0080049

**Published:** 2013-11-14

**Authors:** Salma R. I. Ribeiz, Fabio Duran, Melaine C. Oliveira, Diana Bezerra, Claudio Campi Castro, David C. Steffens, Geraldo Busatto Filho, Cássio M. C. Bottino

**Affiliations:** 1 Old Age Research Group (PROTER), Institute of Psychiatry, University of São Paulo School of Medicine, São Paulo, Brazil; 2 Laboratory of Psychiatric Neuroimaging (LIM 21), Institute and Department of Psychiatry, University of São Paulo, São Paulo, Brazil; 3 Institute of Mathematic and Statistics (IME), University of São Paulo, São Paulo, Brazil; 4 Department of Diagnostic Imaging, Heart Institute (InCor), University of São Paulo School of Medicine, São Paulo, Brazil; 5 Department of Psychiatry, University of Connecticut School of Medicine, Farmington, Connecticut, United States of America; Bellvitge Biomedical Research Institute-IDIBELL, Spain

## Abstract

The relationship between structural changes in grey matter and treatment response in patients with late-life depression remains an intriguing area of research. This magnetic resonance imaging (MRI) study compares the baseline grey matter volume of elderly people with and without major depression (according to the DSM-IV-TR criteria) and assesses its association with antidepressant treatment response. Brain MRI scans were processed using statistical parametric mapping and voxel-based morphometry. The sample consisted of 30 patients with depression and 22 healthy controls. We found a significant volumetric reduction in the orbitofrontal cortex bilaterally in patients in comparison with controls. According to their remission status after antidepressant treatment, patients were classified as remitted or not remitted. Compared with controls, remitted patients showed a volumetric reduction in the orbitofrontal cortex bilaterally and in another cluster in the right middle temporal pole. Non-remitted patients showed an even greater volumetric reduction in the orbitofrontal cortex bilaterally compared with controls. To investigate predictive factors of remission after antidepressant treatment, we used a logistic regression. Both baseline Mini Mental State Examination score and baseline left superior lateral orbitofrontal cortex volume (standardized to the total grey matter volume) were associated with remission status. Our findings support the use of regional brain atrophy as a potential biomarker for depression. In addition, baseline cognitive impairment and regional grey matter abnormalities predict antidepressant response in patients with late-life depression.

## Introduction

Despite the development of new antidepressants (AD) over the past two decades, the odds of achieving remission in patients with depression remain low. Moreover, reliable predictors of treatment response are lacking [1,2]. Approximately 40% of patients with depression do not respond to treatment with the first prescribed AD, and 20% experience chronic depression [3].

These results from studies of young adults with depression also hold true for older patients. Late-life depression (LLD) is defined as a major depressive episode occurring in older adults, and is a heterogeneous mood disorder frequently associated with cognitive impairment. LLD encompasses both late-onset cases (late onset depression, LOD) as well as early-onset cases (early onset depression, EOD) that recur or continue into later years of life [4]. 

Efforts have been made to improve remission rates in LLD. Available data (optimization strategies, drug changes, algorithms, combined and augmentation pharmacological treatments) can guide pharmacological treatment [5] in both the acute and maintenance stages, but further research is required to guide clinical strategies when remission is not achieved. Few biological markers have been correlated with treatment outcomes in patients with LLD. Biological markers of treatment response may include structural brain changes observed via neuroimaging. To investigate network integrity in LLD in vivo, magnetic resonance imaging (MRI) can be an ideal tool.

Grey matter abnormalities within frontal-subcortical and limbic networks are hypothesized to play a key role in the pathophysiology of late-life depression [6,7]. The orbitofrontal cortex (OFC) is known to play a role in emotional and visceral regulation [8]. According to a recent meta-regression analysis [9] of morphometric MRI studies among patients with major depression disorder, depression was associated with volumetric decreases in the prefrontal and orbitofrontal cortices, cingulate cortex, and hippocampus in young adults and older participants [10-12]. However, studies that specifically evaluated patients with LLD compared with controls have found great heterogeneity in terms of grey matter (GM) abnormalities associated with depression. Some studies have not found significant differences in GM volume between older patients with depression and age- matched subjects [13-16]. 

Moreover, a positive correlation exists between the structural brain abnormalities in patients with LLD and their treatment responses, especially in the volumes of the hippocampus [17] and anterior cingulate cortex [18,19]. However, this issue remains controversial as another study [20] did not find any correlation. A recent review of the biological basis of LLD [21] indicated that only a few studies to date have examined how the structural changes observed in patients with LLD influence clinical outcomes [22], and the results are not conclusive. The relationship between GM structural differences and treatment outcomes in patients with LLD remains an intriguing area of research [21]. 

We combined cross-sectional and prospective study design of patients with current LLD to evaluate the influence of depression on their brains and assess the effect of baseline GM volumes on AD treatment response. We hypothesized that baseline regional GM volume would be reduced in patients with LLD depression compared with healthy elderly volunteers and that regional GM reduction would predict AD response.

## Methods

### Ethics Statement

Clinical investigation was conducted according to the principles expressed in the Declaration of Helsinki. The institutional review board, which is entitled CAPPesq (Comissão de Ética para Análise de Projetos de Pesquisa do HC-FMUSP) approved the complete protocol, including the consent procedure. After the study was completely described to all participants, their written informed consent was obtained. Study participants did not receive any monetary compensation.

### Participants

Study participants were selected from two sources: an epidemiologic study of elderly residents of the city of Sao Paulo [23], in which individuals were screened positive for depression (depression scale-D10-score ≥7) [24] and a pool of outpatients who received treatment for depression at the Old Age Research Group (PROTER), Institute of Psychiatry, University of Sao Paulo School of Medicine. 

To be eligible for this study, patients had to be at least 60 years old and meet the DSM-IV-TR [25] criteria for major depressive disorder based on a diagnostic interview administered by trained geriatric psychiatrists (more details are described elsewhere [26]). Control participants were adults who were at least 60 years old without depression from the Department of Geriatrics, University of Sao Paulo School of Medicine. 

Exclusion criteria for the patient group (PG) included a diagnosis of dementia or other organic mental disorder or DSM-IV-TR-criteria-based diagnoses of any psychiatric disorder other than depression (although patients with anxiety disorders comorbid with depression were not excluded). Exclusion criteria for the control group (CG) included current or previous DSM-IV-TR-criteria-based diagnosis of any psychiatric disorder (including depression) or diagnosis of dementia or other organic mental disorders.

### Study procedures

At baseline, a geriatric psychiatrist administered a standardized clinical assessment to all participants that included the Mini Mental State Examination (MMSE) [27]; the CAMDEX and CAMCOG [28], translated and adapted for the Brazilian population [29]; the “Bayer Activities of Daily Living Scale” (B-ADL) [30], adapted for the Brazilian population [31]; the Hamilton Rating Scale for Depression (HAM-D) [32]; the Montgomery-Asberg Depression Rating Scale (MADRS) [33]; the Cumulative Illness Rating Scale (CIRS) [34]; and the Clinical Global Impression (CGI) [35]. Clinical assessments (HAM-D, MADRS and CGI) were repeated every month.

To ensure that participants with early dementia were excluded from the PG, the B-ADL was administered to the informants. According to a previous study of Brazilian patients [31], a cutoff point of B-ADL≥ 3.12 provides adequate sensitivity and specificity and positive and negative predictive values to discriminate patients with mild to moderate dementia from non-demented elderly participants. All depressed individuals were treated according to a modified version of the Duke Somatic Algorithm Treatment for Geriatric Depression [36] over 24 weeks in a prospective cohort design with a follow-up assessment [26]. 

### Remission criteria

We considered patients to be remitted when their MADRS score decreased below 8 during the initial 12 weeks of the study and remained below 8 until the end of the study (24 weeks). All patients whose MADRS scores decreased to 8 but rose above 8 during the study or whose MADRS score remained at 8 or above throughout the study were considered not remitted [26]. 

### MRI acquisition and processing

All MRI scans were acquired at baseline using a 1.5 Tesla scanner (GE Signa Horizon LX; General Electric, Milwaukee, WI, USA) at the Heart Institute (InCor) of the University of Sao Paulo School of Medicine. The MR images were obtained using a T1-weighted sequence (Fast Spoiled Gradient-Echo, 124 coronal slices, slice thickness= 1.2 mm, field of view=256 mm, time repetition (TR) = 6.5 ms, time echo (TE) = 1.49 ms, flip angle=15°). In addition, a whole brain T2-weighted sequence (20 axial slices, slice thickness=6 mm, TR=4500, TE=105 ms, flip angle=90°) was acquired for diagnostic evaluation to rule out any other neurological problems.

The data were processed using the current version of Statistical Parametric Mapping, Version 8 (SPM8, Wellcome Trust Centre of Neuroimaging, London, United Kingdom), implemented in MATLAB R2008a (MathWorks, Sherborn, MA). First, all anatomical images were reoriented; the mm coordinate of the anterior commissure matched the origin _xyz_ (0, 0, 0), and the orientation approximated Montreal Neurological Institute (MNI) space. Then, all images were segmented and classified into GM, white matter and cerebrospinal fluid using the unified segmentation implemented in SPM8, which provides both the native space versions and Diffeomorphic Anatomical Registration using Exponentiated Lie algebra (DARTEL) imported versions of the tissues [37]. A customized template was created from the healthy participants using the DARTEL protocol [38,39]. The deformation field was applied to the segmented images in sequence. Finally, the images created in the previous step were standardized to MNI space, re-sliced to 1.5x1.5x1.5mm voxels and smoothed using a 10-mm FWHM Gaussian kernel. The total GM volumes were obtained from the modulated images.

Anatomical masks that are available within the Anatomical Automatic Labeling (AAL) SPM toolbox were used separately in each hemisphere (left and right, respectively), resulting in search volumes of 12,008 and 12,343 voxels for the OFC; 2,061 and 1,813 voxels for the gyrus rectus; 1,582 and 1,927 voxels for the medial OFC; 2,159 and 2,372 voxels for the middle lateral OFC; 2,157 and 2,238 voxels for the superior lateral OFC; 4,049 and 3,993 voxels for the inferior lateral OFC. These anatomical masks were applied in the patients´ images with the use of “roi_vol_cal.m” tool (https://www.jiscmail.ac.uk/cgi-bin/webadmin?A2=spm;74511e9f.0810) to extract the gyrus rectus and the OFC volume values. Then these volume values were exported to the SPSS software.

### Statistical analysis

#### Sociodemographic and clinical data

All data analyses employed the Statistical Package for the Social Sciences, Version 14 for Windows (SPSS, Inc., Chicago, IL, USA). First, we performed descriptive analyses of the sociodemographic and clinical characteristics (frequencies and percentages). We employed the chi-squared test to compare categorical data and either Student’s t-test or the Mann-Whitney U test for continuous data. The level of significance was set at 0.05. A stepwise-forward logistic regression was used to address whether baseline characteristics predict AD treatment response. 

#### Neuroimaging data

Between group regional differences in GM volumes were assessed using the general linear model in SPM8, and the significance of each effect was estimated from the distributional approximations of Gaussian random fields [40]. An absolute threshold mask of 0.05 was used for GM analyses. Total grey matter volume (measured with SPM8) was entered into the design matrix as nuisance covariate. Results were assessed using the family-wise error (FWE) threshold of P_FWE_ ≤ 0.05, corrected for multiple comparisons. In all analyses, we converted MNI coordinates of voxels of maximal statistical significance to the Talairach and Tournoux system [41,42].

## Results

### Sample characteristics

The final sample consisted of 30 patients and 22 controls. In the PG, 21 (70%) patients were classified as having late-onset depression (i.e., the onset of major depressive symptoms occurred after 60 years old, LOD), and 9 (30%) patients were classified as having early-onset depression (EOD). The mean number of previous depressive episodes was 1.48 (SD= 2.26). Importantly, however, 12 patients reported previous or current use of AD, and 4 patients were taking an AD at baseline. 

We compared the sociodemographic and clinical characteristics of the PG and the CG. All participants were right-handed. No differences were found with regard to age or gender (Table 1). There were significant differences between the PG and the CG in terms of mean years of education. Specifically, the CG had more education (in years) compared with the PG (PG: mean=6.50, SD=5.49; CG: mean=9.91, SD=5.12; t=-2.27; p=0.027). However, across the RG, NRG and CG no significant difference (p=0.07) was found with regard to education. Although remission rates of the LOD group was lower (42.9%) than the EOD group (66.7%), there was no significant statistical difference between groups (p=0.427). 

**Table 1:** Baseline socio-demographic and clinical characteristics of the sample.

	**Patients (n=30)**	**Controls (n=22)**	**Statistical test and p-value**
**Gender Female (%)**	23 (76.7%)	17 (77.3%)	X^2^=0.003, p=0.959
**Age in years (SD)**	70.73 (6.59)	70.41 (7.58)	t=0.164, p=0.87
**Education in years (SD)**	6.50 (5.49)	9.91 (5.12)	t=-2.27, **p=0.027**
**MMSE (SD)**	24.90 (4.188)	27.95 (1.84)	t= -3.196, **p=0.002**
**CAMCOG (SD)**	81.87 (15.469)	91.95(7.18)	t=-2.837, **p=0.007**
**HAM-D (SD)**	19.37 (5.951)	2.27 (1.69)	t=13.059, **p< 0.001**
**MADRS (SD)**	24.10 (8.519)	1.18 (1.33)	t=12.475, **p< 0.001**
**B-ADL (SD)**	2.44 (1.133)	1.28 (0.59)	MW= 108; **p< 0.001**
**CIRS-Severity (SD)**	1.29 (0.176)	1.17 (0.15)	t=2.495; **p=0.016**
**CIRS-Comorbidity (SD)**	0.87 (0.860)	0.40 (0.82)	MW= 228; p=0.067
**Age at onset of depression (%)**	EOD= 9 (30%) LOD=21 (70%)	-------------	------------

Values in boldface are significant. N= number of patients; MMSE= Mini Mental State Examination; CAMCOG= Cambridge Cognitive Examination; B-ADL= Bayer Activities of Daily Living Scale; MADRS= Montgomery-Asberg Depression Rating Scale; HAM-D= Hamilton Rating Scale for Depression; CIRS= Cumulative Illness Rating Scale; EOD= Early Onset Depression; LOD= Late Onset Depression; SD= standard deviation; X^2^ refers to Chi-square; t refers to T test; MW refers to Mann-Whitney U. Gender and age at onset of depression were described in frequency and percentage.

The PG had significantly lower scores on the MMSE and CAMCOG and significantly higher scores on the CIRS-severity, B-ADL, MADRS and HAM-D compared with the CG. Cut-off values for the MADRS scores [43] were used to classify different grades of severity of depression: mild (7-19), moderate (20-34), severe (35-60). In the sample studied, 13.3% (n= 4) of patients had severe symptoms, 56.6% (n= 17) had moderate and 30% (n=9) had mild depressive symptoms at baseline. The CIRS-comorbidity scores did not differ between PG and CG. Cumulatively, the most common illnesses in the PG and CG were hypertension and musculoskeletal diseases. 

Voxel-based morphometry (VBM) – DARTEL

With regard to the total GM volume (mL) between PG (mean= 630.788, SD= 45.377) and CG (mean= 622.898, SD= 54.625, p= 0.604), we did not find statistically significant differences. 

**Figure 1:**
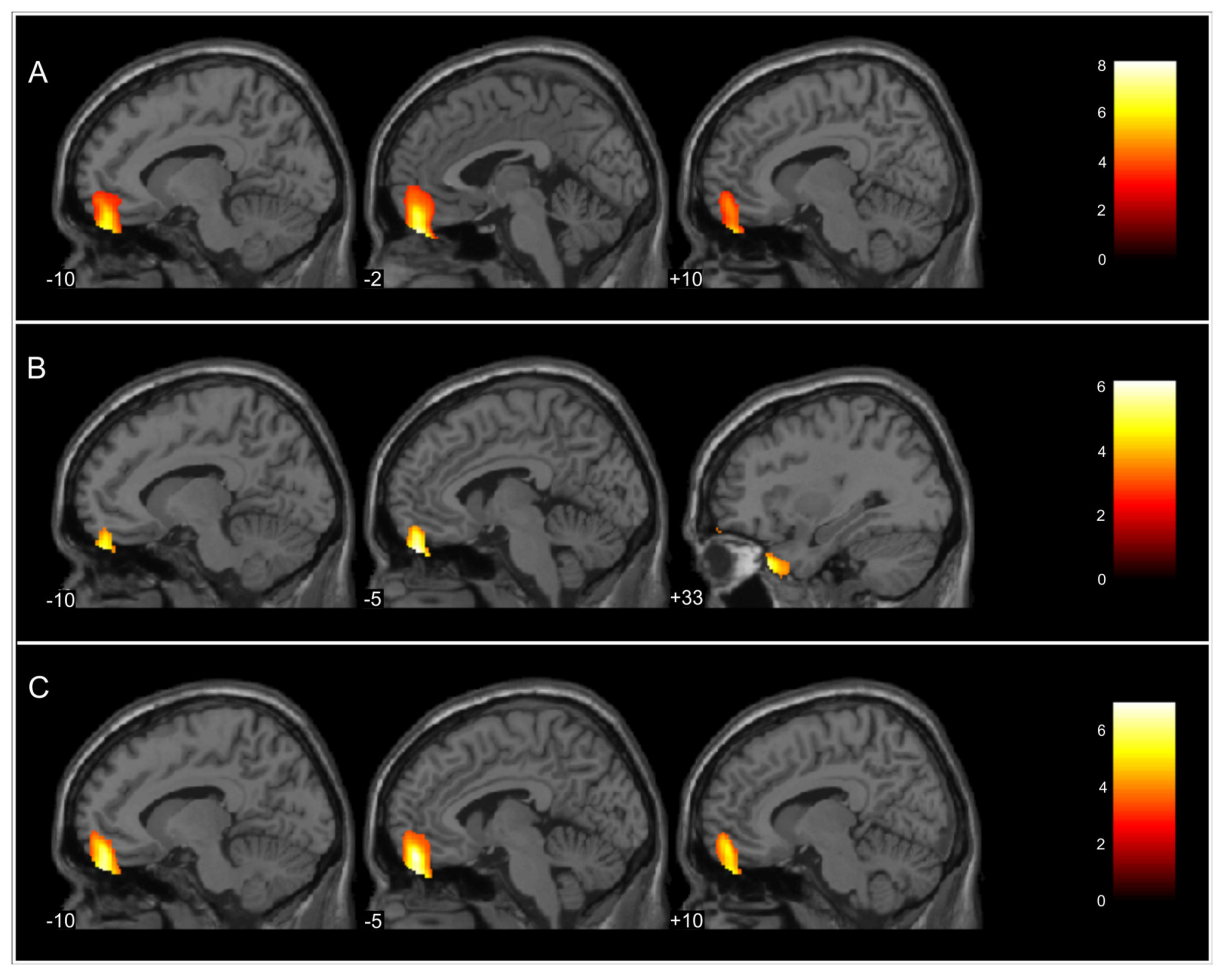
Grey matter volume decrease in patients with late life depression. (A) Cluster of decreased grey matter volume in the patient group (n=30) relative to the control group (n=22). (B) Clusters of decreased grey matter volume in the remitted group (n=15) relative to the control group. (C) Cluster of decreased grey matter volume in the non-remitted group (n=15) relative to the control group. Brain regions with foci that show significant differences (yellow; Z>3.09 cutoff, corresponding to *p* < 0.001, uncorrected for multiple comparisons) with regard to grey matter volumes between groups. The foci of significance were overlaid on sagittal brain slices that were spatially normalized to approximate the Talairach and Tournoux stereotaxic atlas [41]. The numbers associated with each frame represent the standard x-axis coordinates.

For whole brain analysis of PG and CG, because the mean years of education significantly differed between patient and control groups (p= 0.027), this variable was included as a covariate in this analysis. We found a statistically significant GM decrease in the PG in comparison with the CG in a cluster (4,927 voxels; peak level _xyz_= -3 48 -30; BA= 11; Z= 6.40; peak voxel p-FWEcorr≤0.001; Figure 1A) that included the gyrus rectus bilaterally and the OFC bilaterally (the medial OFC bilaterally, the middle lateral OFC bilaterally, and the superior lateral OFC bilaterally). 

In whole-brain analyses among RG, NRG, and CG, corrected for multiple comparisons (p-FWEcorr˂ 0.05), we found that the gyrus rectus bilaterally and the OFC bilaterally (the superior lateral OFC bilaterally, the medial OFC bilaterally and the middle lateral OFC bilaterally) are different between the three groups (3,188 voxels; peak level _xyz_ = -6 51 -29; BA=11; Z= 5.76; peak voxel p-FWEcorr≤ 0.001). Afterwards, we proceeded with paired comparisons (RG with CG, NRG with CG, RG with NRG) to better understand the volumetric differences between groups.

 Compared with CG, the RG showed a significant reduction in two clusters of voxels. One of them (1,181 voxels; peak level _xyz_= -5 51 -29; BA= 11; Z= 5.24; peak voxel p-FWEcorr= 0.002) involved the gyrus rectus bilaterally and the OFC bilaterally (the superior lateral OFC bilaterally) and the other cluster included the right middle temporal pole (828 voxels; peak level _xyz_= 33 24 -36; BA= 38; Z= 4.72; peak voxel p-FWEcorr= 0.023; Figure 1B). Likewise, the NRG showed a significant reduction (5,601 voxels; peak level _xyz_= -5 51 -20; BA=11; Z= 5.76; peak voxel p-FWEcorr≤ 0.001) in the gyrus rectus bilaterally and the OFC bilaterally (the medial OFC bilaterally, the middle lateral OFC bilaterally, and the superior lateral OFC bilaterally) compared with the CG (Figure 1C). We did not find significant GM volumetric difference between RG and NRG. 

We used logistic regression to predict remission after AD treatment. Initially, patients’ baseline characteristics and volumetric measures (standardized to the total grey matter volume) of the right, left and both sides of the OFC were used to predict remission after AD treatment. We used the stepwise forward (Wald) method and only baseline MMSE score (p=0.039) could predict clinical outcome. 

Given that standardized left OFC was the last variable excluded from the logistic regression model, we investigated whether a specific OFC subregion could predict remission. The same patients’ baseline characteristics and the volumetric measures (standardized to the total grey matter) of the OFC subregions were used to predict remission after treatment (Table 2). Using the stepwise forward (Wald) method, only baseline MMSE score (p=0.035) and the left superior lateral OFC (LSOFC) volume (p=0.048) could sufficiently classify patients based on the remission criterion (Table 2). According to this model, 73.3% of the patients who remitted and 80.0% of patients who did not remit were correctly classified, for an average of 76.7% for the entire sample (Table 3). 

**Table 2:** Logistic regression model.

**Baseline characteristics**	**Coefficient estimate**	**Standard error**		**Wald χ2**	**Df**	**p value**
**MMSE scores**	0.258		0.122	4.462	1	**0.034**
**Standardized left superior lateral OFC volumes**	2207.256		1118.085	3.897	1	**0.048**
**Constant**	-15.832		6.377	6.164	1	0.013

Baseline characteristics included in the logistic regression: gender; age; education; type of depression (early or late onset depression); MMSE (Mini Mental State Examination) and MADRS (Montgomery-Asberg Depression Rating) scale scores; and standardized volumetric measures of the right and the left gyrus rectus, the medial OFC, the middle lateral OFC, the superior lateral OFC, the inferior lateral OFC.

**Table 3:** Correct classifications of the not remitted group (NRG), remitted group (RG) and the overall percentage based on the baseline standardized left superior lateral OFC volumes and MMSE scores.

**Step 1**	**NRG**	**RG**	**Percent correct**
**NRG**	12	3	80.0%
**RG**	4	11	73.3%
**Overall percentage**	16	14	76.7%

Given the importance of age of onset in studies of late life depression, we examined remission rates taking into account age of onset. Although remission rates of the LOD group was lower (42.9%) than the EOD group (66.7%), the difference was not statistically significant (p=0.427). The small sample size in LOD and EOD groups precluded further modeling.

## Discussion

The results confirmed our primary predictions that we would observe GM abnormalities in LLD. We found OFC volumetric reduction in LLD depression and it predicted AD response. 

The orbitofrontal cortex occupies the ventral surface of the frontal part of the brain [44]. In terms of its neuroanatomical connectivity, the orbitofrontal cortex is uniquely placed to integrate sensory and visceral motor information to modulate behavior through both visceral and motor systems. This has led to the proposal that the orbitofrontal cortex is an important part of the networks that are involved in emotional processing. A recent meta-analysis [45] showed that there is a medial–lateral distinction in the human orbitofrontal cortex, such that activity in the medial orbitofrontal cortex is related to the monitoring, learning and memory of the reward value of reinforcers, whereas lateral orbitofrontal cortex activity is related to the evaluation of punishers that can lead to a change in behavior. The recent convergence of findings from neuroimaging, neuropsychology and neurophysiology indicates that the human orbitofrontal cortex is an important nexus for sensory integration, emotional processing and hedonic experience. It has become clear that the orbitofrontal cortex has an important role in emotional disorders such as depression [44].

Studies examining depressed elderly populations have consistently found smaller OFC volumes [10,12,46,47]. Recently, a meta-analysis confirmed previous findings of GM atrophy in elderly patients with depression [48]. Nevertheless, a recent multimodal study did not find differences in GM volumes between people with LLD and controls [15]. It is important to note, however, that their results might have been influenced by the low HAM-D scores of the LLD group and the fact that most participants were currently using psychotropic medications [15]. 

The presence of GM reduction in specific OFC subregions of PG compared with CG might reflect disease-specific modifications of the elderly people brains with depression [12], which suggests that GM reduction is a characteristic of LLD depression and a potential biomarker for depressive episodes. However, whether this GM reduction occurs before the development of depression (as a pre-existing, vulnerability marker for depression) or is a consequence of depression remains unclear [49]. Sustained exposure to elevated glucocorticoid levels via chronic stress might produce OFC atrophy and make patients vulnerable to major depression [50]. Alternatively, vascular lesions might decrease GM volume [10]. Longitudinal studies are needed to elucidate this issue.

Another interesting finding is related to the OFC volume and outcome prediction: NRG had the smallest OFC volume, followed by RG, and by CG. In comparison with CG, RG showed GM reduction in a cluster that included the bilateral OFC and in another cluster that included the right middle temporal pole. Furthermore, the NRG showed an even greater GM reduction in the OFC bilaterally compared with the CG. More specifically, in comparison with the CG, the NRG showed significant volumetric reductions in some OFC subregions (the right superior lateral OFC, the middle lateral OFC bilaterally, and the medial OFC bilaterally) that RG did not show. 

These results suggest that regional GM reductions can predict treatment response. Other studies identified GM reduction of the cingulum [19] and the hippocampus [17] as predictive factors of outcome. Gunning et al. [19] found an association between the dorsal and rostral anterior cingulate volumes and poorer treatment response to escitalopram in individuals with LLD compared with controls. Hsieh et al. [17] reported that smaller hippocampal volumes were related to decreased response to AD, whereas Janssen et al. [20] did not find structural differences between remitted and non-remitted individuals with LLD. Recently, a study showed that baseline hippocampal volume and frontal pole thickness differed between patients who achieved remission and patients who did not [51]. In addition, they found that primary hippocampal volume and some cognitive variables predict antidepressant response. In the present study, we did not find hippocampal volumetric differences when comparing PG and CG, RG and CG, NR and CG, nor NR and RG. A possible explanation for these results may be due to methodological differences, such as sample size and differences in population characteristics.

Interestingly, we found that the RG had smaller volume in the right middle temporal pole than CG. A previous study found smaller volumes of the right superior frontal cortex, the left postcentral cortex, and the right middle temporal gyrus in remitted geriatric patients relative to controls [52]. There is evidence that the OFC receives projections from temporoparietal cortices which contribute to integrating viscerosensory information with affective signals [53,54]. In depressed patients, three previous MRI studies did not show a difference in total temporal lobe volume compared with controls [55-57]. Moreover, another study [58] compared LLD and control group regarding 24 ROIs. After controlling for multiple comparisons, subjects with LLD had significantly smaller volumes than non-depressed subjects in 17 of 24 areas, which included the OFC, the hippocampus, the parahippocampal area, and the temporal lobe (including the temporal pole). These findings should, however, be viewed cautiously. Another LLD study [46] found prominent brain size reductions in the depressed subjects in the OFC bilaterally. However, cortical GM measurements revealed significant GM increases in the OFC, adjacent to focal trend level significant decreases of GM in the same region. Depressed patients also exhibited significant GM increases in the parietal cortices, as well as the left temporal cortex [46]. This finding warrants further investigation to clarify this issue.

Moreover, the logistic regression (Table 2) showed that baseline MMSE scores and standardized left superior lateral OFC volumes could classify patients based on the remission criteria (Table 3). Previously [26], we found baseline MMSE score as a predictor of poorer treatment response. In the present study, according to the logistic regression model, if we consider the mean values ​​of the variables, the likelihood of remission is 50%. If we increase one unit in the MMSE score, the likelihood of remission will increase 12%. On the other hand, if we increase 8% (range of the confidence interval) the baseline standardized LSOFC volume, the likelihood of remission will increase 36%. These results suggest that depressed elderly patients with cognitive impairment and with smaller LSOFC volume will have a worse outcome of depression treatment.

Our data supports the hypotheses that LLD is associated with smaller OFC volume and that smaller OFC can be a potential biomarker of LLD. Moreover, we found that a general cognitive evaluation (MMSE) and a volumetric measure of the OFC can be potential biomarkers of treatment response. 

### Strengths

This cross-sectional and prospective clinical and neuroimaging study examined elderly patients with depression. Our sample consisted of patients with mild to moderate symptoms of depression without psychotic symptoms (which represents the majority of patients with depression in the community). Most of these patients did not use AD during their MRI brain scan (at baseline), thus partially avoiding the confounding neurogenic effects of the AD on the brain. Patients and controls generally had few and low severity comorbidities. The low education level of this sample is representative of elderly patients in developing countries.

### Limitations

Our ﬁndings should be interpreted with caution due to our relatively small sample size, although our significant finding is noteworthy for such a relatively small sample. Furthermore, our sample is composed of patients with mild to moderate symptoms of depression; thus, we may not generalize these findings to patients with more severe depression or to patients who have been hospitalized. The small sample size also precluded an analysis of age of depressive onset that would allow us to examine the effect of age of onset in our models. We were unable to examine other factors that increase vulnerability for depression (e.g., genetic markers, a history of abuse or stressful early life events) that might be related to volumetric reduction that precedes depression onset. 

Moreover, our results cannot shed light on the causality between LLD and GM volumetric reduction due to its cross-sectional design. To elucidate the relationship between brain changes and the pathophysiology of LLD, longitudinal neuroimaging studies are required. 

## Conclusions

GM reduction in the OFC bilaterally is a characteristic of LLD and a potential biomarker of this condition. Baseline cognitive impairment and smaller left superior lateral OFC volume can predict outcome after AD treatment in LLD.
